# Genomic Prediction of Sunflower Hybrids Oil Content

**DOI:** 10.3389/fpls.2017.01633

**Published:** 2017-09-21

**Authors:** Brigitte Mangin, Fanny Bonnafous, Nicolas Blanchet, Marie-Claude Boniface, Emmanuelle Bret-Mestries, Sébastien Carrère, Ludovic Cottret, Ludovic Legrand, Gwenola Marage, Prune Pegot-Espagnet, Stéphane Munos, Nicolas Pouilly, Felicity Vear, Patrick Vincourt, Nicolas B. Langlade

**Affiliations:** ^1^LIPM, Université de Toulouse, INRA, Centre National de la Recherche Scientifique Castanet-Tolosan, France; ^2^Terres Inovia, AGIR Castanet-Tolosan, France; ^3^GDEC, INRA, Université Clermont II Blaise Pascal Clermont-Ferrand, France

**Keywords:** genomic selection, factorial design, sunflower, oil content, hybrid, GBS

## Abstract

Prediction of hybrid performance using incomplete factorial mating designs is widely used in breeding programs including different heterotic groups. Based on the general combining ability (GCA) of the parents, predictions are accurate only if the genetic variance resulting from the specific combining ability is small and both parents have phenotyped descendants. Genomic selection (GS) can predict performance using a model trained on both phenotyped and genotyped hybrids that do not necessarily include all hybrid parents. Therefore, GS could overcome the issue of unknown parent GCA. Here, we compared the accuracy of classical GCA-based and genomic predictions for oil content of sunflower seeds using several GS models. Our study involved 452 sunflower hybrids from an incomplete factorial design of 36 female and 36 male lines. Re-sequencing of parental lines allowed to identify 468,194 non-redundant SNPs and to infer the hybrid genotypes. Oil content was observed in a multi-environment trial (MET) over 3 years, leading to nine different environments. We compared GCA-based model to different GS models including female and male genomic kinships with the addition of the female-by-male interaction genomic kinship, the use of functional knowledge as SNPs in genes of oil metabolic pathways, and with epistasis modeling. When both parents have descendants in the training set, the predictive ability was high even for GCA-based prediction, with an average MET value of 0.782. GS performed slightly better (+0.2%). Neither the inclusion of the female-by-male interaction, nor functional knowledge of oil metabolism, nor epistasis modeling improved the GS accuracy. GS greatly improved predictive ability when one or both parents were untested in the training set, increasing GCA-based predictive ability by 10.4% from 0.575 to 0.635 in the MET. In this scenario, performing GS only considering SNPs in oil metabolic pathways did not improve whole genome GS prediction but increased GCA-based prediction ability by 6.4%. Our results show that GS is a major improvement to breeding efficiency compared to the classical GCA modeling when either one or both parents are not well-characterized. This finding could therefore accelerate breeding through reducing phenotyping efforts and more effectively targeting for the most promising crosses.

## 1. Introduction

Sunflower is one of the main oilseed crops worldwide. Although this crop was domesticated in North America, the sunflower was developed as a major crop in Russia in the first half of the twentieth century, when the breeding programs of V.S. Pustovoit increased the seed oil content from 25–30 to 45–50%. This success largely reflected the high heritability of this key breeding trait. Based on two segregating populations, involving wild-type and improved germplasms, Fick (1975) provided the first estimation of the narrow-sense heritability of seed oil content as 0.52–0.61, suggesting that the contribution of genetic additive variance is prominent. As the seed oil content can now be rapidly and inexpensively measured using nuclear magnetic resonance (NMR), selection can be performed in segregating progenies and on a single plant basis, from the F2 generation onwards. Vear et al. (2010) indicated that hybrids generally show heterosis for oil content, which is not typically the case when the parents contain approximately 50% oil.

Mapping quantitative trait locus (QTL) for oil content was initiated more than 20 years ago (Leon et al., 1995), providing congruent results across the segregating populations involved (Mestries et al., 1998; Bert et al., 2002; Bachlava et al., 2010; Merah et al., 2012) and confirming both the quantitative nature of the trait (several loci) and its high heritability (mapped QTLs accounted for 10 to 51% of the phenotypic variability). The high level of heritability for oil content suggests that this trait is an easy character to breed for, and the absence of important interactions with environmental conditions makes it feasible to obtain valid general conclusions as to the interest of a genotype for this character based on a small number of measurements under different conditions. Thus, there is no direct requirement for genomic studies to replace phenotypic measurements. However, because robust oil content data are easy to obtain, oil content is a good model trait to test the power of genomic selection models prior to applying these models to explore more complex characters, such as seed yield or quantitative resistance to fungal diseases.

Genomic prediction refers to the prediction of genetic value based on markers spread throughout the entire genome. In this framework, a mathematical model is trained on past genotyped and phenotyped resources, and new unobserved individuals who are genotyped but not phenotyped are predicted with this learned model. Among the different models since the work of Meuwissen et al. (2001), the mixed model and the genome-wide best linear prediction (GBLUP) of unobserved individuals proposed by VanRaden (2008) is the most popular model. Originally, the mixed model of Meuwissen et al. (2001) assumes that the haplotype effects of all genomic regions follow the same Gaussian distribution. When limiting each genomic region to a single marker, this model is known as the ridge regression BLUP (RR-BLUP). The RR-BLUP and GBLUP models are equivalent models (Endelman, 2011) and Goddard (2009) showed that they are similar to the classical pedigree mixed model when relatedness between individuals are estimated with markers. Mixed models and BLUP have been comprehensively compared to other methods of genomic prediction as penalized regressions (Li and Sillanpää, 2012, for a review), Bayesian modeling (Kärkkäinen and Sillanpää, 2012, for a review), semi-parametric learners as the reproducing kernel Hilbert space (RKHS) (Gianola et al., 2006) and non-parametric methods, such as random forest (Chen and Ishwaran, 2012). Depending on the trait studied, one or the other of these methods was demonstrated as more reliable, but the best performers provided comparable accuracies (Heslot et al., 2012; Haws et al., 2015). The mixed model framework has consistently produced comparative results to those obtained with more complicated models. The simplicity, efficient computer implementation and flexibility of this model have meant that most novel modeling ideas have been based on this framework.

As previously described, in GBLUP modeling, effects of genetic markers are assumed to follow the same Gaussian distribution. This unrealistic assumption does not consider the biological mechanisms underlying phenotypic variation. Speed and Balding (2014) proposed an extension of GBLUP, called MultiBLUP, to include multiple random effects allocated to different sets of SNP markers. The close variants are grouped and the relatedness of random genetic effects is determined for each set using a similarity matrix calculated using the SNPs of the region of interest, thus modeling a different effect-size distribution for each set. Using MultiBLUP, Wolfe et al. (2016) predicted disease resistance in cassava. Delimiting the genome to a region representing between 30 and 66% of the genetic resistance and using the remaining SNPs facilitated an increase in the precision of prediction from 0.53 to 0.58 compared to GBLUP. Similarly, Sarup et al. (2016) using genomic feature BLUP, which is equivalent to MultiBLUP, asserted using several porcine traits that MultiBLUP prediction accuracy is better than GBLUP when the set of SNPs linked to previously known QTLs explained more than 10% of trait variability. Other methods for integrating information a priori have also been tested. Zhang et al. (2014) proposed the consideration of QTLs based on assigning a predefined weight to the region of interest in the relatedness. These QTLs could also be included as fixed effects (Bernardo, 2014; Spindel et al., 2016). Prediction accuracy increases up to 30% depending on the trait when SNPs are derived from a GWAS performed with the data used to train the genomic model (Spindel et al., 2016). However, the integration of SNPs from the literature does not show the same ability to improve on the model accuracy. MultiBLUP is currently included in the framework of multi-kernel mixed models (Weissbrod et al., 2016), as are included linear mixed models for complex trait architecture (dominance and epistasis) (de los Campos et al., 2009).

Mixed models for hybrid predictions based on GCA and/or specific combining ability (SCA) have long been applied prior to the use of genetic markers. In maize, Bernardo (1996) enhanced this old model by proposing the pedigree BLUP model, which uses co-ancestry coefficients between parents of hybrids. First attempt to estimate these co-ancestry coefficients using molecular markers was proposed in Schrag et al. (2006) and further generalized by Technow et al. (2014) using whole genomic data. In sunflower, Reif et al. (2013) did not observe any improvement of genomic BLUP compared to the pedigree BLUP. Equality between the two approaches was consistent with the work of Goddard (2009), as Reif et al. (2013) estimated co-ancestry coefficients using the same markers included in the genomic BLUP, so the two predictions are equivalent. In contrast to Reif et al. (2013), we want to compare prediction accuracy of hybrid genetic values using a classical mixed model that makes use of only pedigree information, if available, to other mixed models that use genomic data to compute relatedness between hybrids and parents. We make this comparison using seed oil content phenotypes observed in an incomplete factorial design produced in the course of the SUNRISE project.

## 2. Materials and methods

### 2.1. Plant materials

Hybrids were obtained as an incomplete factorial design by crossing 36 maintainer lines with 36 restorer lines. The complete hybrid panel contained 492 hybrids. These plants were sown in 11 different environments (5 different environments in 2013, 3 different environments in 2014, and 3 different environments in 2015) (Bonnafous et al., 2017), but for the present study of oil content, we discarded 2 environments due to imperfect randomizations and inaccurate phenotypic observations.

The parents were genotyped by sequencing using the XRQ genome as the reference parent, and their genotypes were imputed by chromosome using Beagle (Browning and Browning, 2009) as described in Badouin et al. (2017). SNPs that were not polymorphic in either the maintainer or the restorer panels were discarded, and a single referent SNP was maintained, representing each set of redundant SNPs (i.e., SNPs in complete linkage disequilibrium in the 72 parent panel). Finally, the genomic data comprised 468,194 non-redundant SNPs, and hybrid genotypes were deduced from the parent genotypes.

Measurement of oil seed content was observed by NMR using a minispec (MQ10H, mq Series, version 1.2, January 2000, Bruker, Germany). Each 20-ml seed sample was first dried for 24 h at 80°C and subsequently analyzed at room temperature.

Genes related to oil metabolism have been identified through the metabolic network reconstruction of the genome annotation of the sunflower (Badouin et al., 2017). The oil metabolism super-pathway has been manually constructed from several inferred metabolic pathways. Relations between genes and reactions were automatically inferred and curated based on the literature. Further details are provided in the on-line materials Badouin et al. (2017), and the examined genes are listed in the Supplementary material (Data sheet S1). An interactive view of the pathway showing with the gene/reaction links is available at https://pathway-tools.toulouse.inra.fr/HANXRQ/NEW-IMAGE?type=PATHWAY&object=PWY198A-2 We considered all SNPs identified in the genes listed above, and we added all the SNPs located 1,000 bases upstream and downstream of these genes.

### 2.2. Predictions of hybrid performances

The phenotypes were initially adjusted using a spatial model, including the line and column numbers in the field, the repetition when necessary and the genotype status (check variety or hybrid) as fixed factors, and a random independent effect modeling the genotypic value of observed individuals completed the model as described in Bonnafous et al. (2017).

Predictions of hybrid performance were computed based on BLUP using several linear mixed models within each environment. Variance components of linear models were estimated using restricted maximum likelihood (REML) with the ASReml-R package (Butler et al., 2007). The models are similar to the progeny models described in Bouvet et al. (2016).

#### 2.2.1. GCA-based prediction

The hybrid genetic value of the *fm* hybrid was predicted using ${\hat{GCA}}_{f}+{\hat{GCA}}_{m}$, where *f* denoted the female line and *m* the male line. GCA BLUPs were obtained using the following model:

(1) $$y_{fm}=\mu +GCA_{f}+GCA_{m}+\epsilon_{fm}\;(GCA\text{ model})$$

where *y*_*fm*_ is the adjusted phenotype in an environment, μ is the mean, *GCA*_*f*_ and *GCA*_*m*_ are the random GCA effects of female *f* and male *m*, respectively, and ϵ_*fm*_ denotes error. All random effect are assumed Gaussian and independent with $\sigma_{GCA_{f}}^{2}$, $\sigma_{GCA_{m}}^{2}$, and $\sigma_{\epsilon }^{2}$ for the GCA female, GCA male, and residual variances, respectively. When the parent pedigree is known, the relatedness of parents can be included in the variances of *GCA* random effects using a coancestry coefficient matrix in this model. However, the pedigree of the parental lines was considered to have too much uncertainty to account for using this analysis. Moreover, parents of the factorial design were chosen to be as unrelated to provide a good representation of the core collection studied in Cadic et al. (2013). Therefore, these parental lines are assumed independent.

#### 2.2.2. *FM* and *FMI* model predictions

The hybrid genetic value of the *fm* hybrid was predicted using BLUPs of ${\hat{F}}_{f}+{\hat{M}}_{m}$ in the *FM* model and ${\hat{F}}_{f}+{\hat{M}}_{m}+{\hat{I}}_{fm}$ in the *FMI* model.

(2) $$y_{fm}=\mu +F_{f}+M_{m}+\epsilon_{fm}\;(\text{FM model})$$

(3) $$y_{fm}=\mu +F_{f}+M_{m}+I_{fm}+\epsilon_{fm}\;(\text{FMI model})$$

where *F*_*f*_, *M*_*m*_, and *I*_*fm*_ are the random effects of female *f* and male *m* lines and their interactions, respectively, and ϵ_*fm*_ denotes error. Let ***F***, ***M***, ***I***, and **ϵ** denote vectors of female, male, interaction and error residual effects, respectively. $F\sim N\left(0,\sigma_{f}^{2}K_{f}\right)$, $M\sim N\left(0,\sigma_{m}^{2}K_{m}\right)$, $\text{I}\sim N\left(0,\sigma_{fm}^{2}K_{\mathrm{fm}}\right)$, $\epsilon \sim N\left(0,\sigma_{\epsilon }^{2}\mathrm{Id}\right)$ where ***K***_***f***_ is the kinship matrix for females; ***K***_***m***_ is the kinship matrix for males; ***K***_***fm***_ is the kinship matrix for the interaction between males and females; and $\sigma_{f}^{2}$, $\sigma_{m}^{2}$, $\sigma_{fm}^{2}$ and $\sigma_{\epsilon }^{2}$ are female, male, female by male interaction and residual variance, respectively.

$K_{f}=X_{f}X_{f}^{\prime }$, $K_{m}=Z_{m}Z_{m}^{\prime }$, and $K_{\mathrm{fm}}=W_{\mathrm{fm}}W_{\mathrm{fm}}^{\prime }$ with ***W***_***fm***_ as the Hadamard product between ***X***_***f***_ and ***Z***_***m***_, where ***X***_***f***_ is the vector of $x_{f}^{l}$, the centered (0 or 1) allele transmitted by female *f* at the *l*th marker locus, and ***Z***_***m***_ is the vector of $z_{m}^{l}$, the centered (0 or 1) allele transmitted by male *m* at the *l*th marker locus.

Note that the *GCA* model and the *FM* model differ only by the assumptions made on the variance-covariance of the random parental effects. The variance-covariance matrix of these parental effects was proportional to the identity matrix in the *GCA* model when it was computed using markers in the *FM* model. Both models predict the parental GCA and the hybrid prediction is the sum of predicted parental GCA.

We performed two *FM* models: (i) in one model, the parental design matrices (*X_f_* and *X_m_*) were computed including 468,194 genome SNPs, (ii) in the other model, these matrices included only a pre-selected set of SNPs in genes previously demonstrated as involved in the oil content metabolism network.

#### 2.2.3. Multi-kernel model predictions

MultiBLUP was proposed by Speed and Balding (2014) in trait additive modeling. This model was further extended to consider more complex trait architecture, such as epistasis, and this model was included in the general and highly flexible framework of the multi-kernel model (Weissbrod et al., 2016). In the simplest linear additive form of Speed and Balding (2014), these models comprise several additive random effects, each with its own kinship (linear kernel) and variance. These models can easily be generalized to *FM* or *FMI* models by modeling several groups of parental random factors, each group having its own kinship and variance. The hybrid genetic value is subsequently predicted based on the sum of the BLUP values for the female and male effects in different groups in the *FM* model, as an example.

We performed two multi-kernel BLUP models using female and male SNP allelic effects. One prediction is the generalization of MultiBLUP to *FM* model using two SNP groups, with the SNPs in genes or close to genes involved in the oil content metabolism network in one group, and all remaining SNPs in the other group.

The other multi-kernel model adds to female and male kinships, two epistasis parental kinships computed using the Hadamard product ${K_{f}}^{*}K_{f}$ and $K_{m}^{*}K_{m}$ for the femalexfemale and malexmale epistasis kinship, respectively. This model is a generalization of additivexadditive epistasis modeling proposed by Su et al. (2012) to *FM* model. The Su et al. (2012) epistasis modeling was demonstrated to explicitly model all pairwise additivexadditive SNP interactions by Jiang and Reif (2015) and is similar to the model of Bouvet et al. (2016).

#### 2.2.4. Predictive ability of hybrid performances

Predictive ability or phenotypic accuracy of predictions was based on the Pearson's correlation between the observed phenotypes and their predicted values for hybrids that were not used to train the models, the so-called test individuals or out-of-population hybrids. This accuracy was computed as the mean of 100 test sets. We used two sampling schemes: a random draw of 10% of the hybrids or a random draw of 10% of the parent lines for which all observed descendants were included in the test set. This latter sampling enables the generation of test sets comprising only T1 or T0 hybrids, consistent with Technow et al. (2014), i.e., out-of-population samples with parents never observed through hybrid progeny. As for the SUNRISE incomplete factorial design, all parents had a nearly equal number of descendants, this sampling scheme generated approximately 10% of hybrids.

## 3. Results

**Figure 1:**
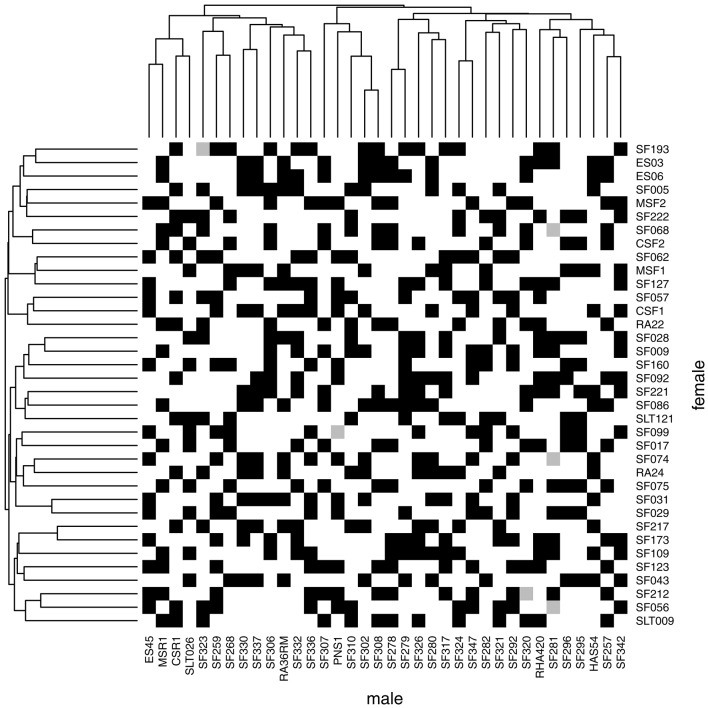
Incomplete factorial design of SUNRISE from 36 maintainer lines (females, in column) and 36 restorer lines (males, in row). Black squares indicate the 486 hybrids with phenotypic observations on at least one environment and gray squares indicate hybrids that were planned but were never observed for oil content in the MET. Male and female dendrograms are based on their kinship matrices.

Figure 1 presents the SUNRISE incomplete factorial design with females and males arranged according to a hierarchical clustering based on VanRaden's kinship matrices (VanRaden, 2008). As part of the sunflower elite collection studied by Cadic et al. (2013), male parents are restorers of the CMS PET1 cytoplasmic male sterility, [R-lines] for which female parents are maintainers, [B-lines]. Male parents seem slightly more structured and related than female parents, consistent with the findings of Cadic et al. (2013), who distinguished two main subgroups in the B-germplasm among the core collection. The factorial design was completely connected and almost balanced, as the parents were involved in nearly an equal number of crosses. Among the 492 hybrids generated, 486 hybrids were observed in the MET at least once for the oil content phenotype; thus, for this trait, parents were observed for a minimum of 12 to a maximum of 15 descendants (number of observed hybrids per parents in the MET are detailed in Supplementary Material, Tables S1, S2).

**Figure 2:**
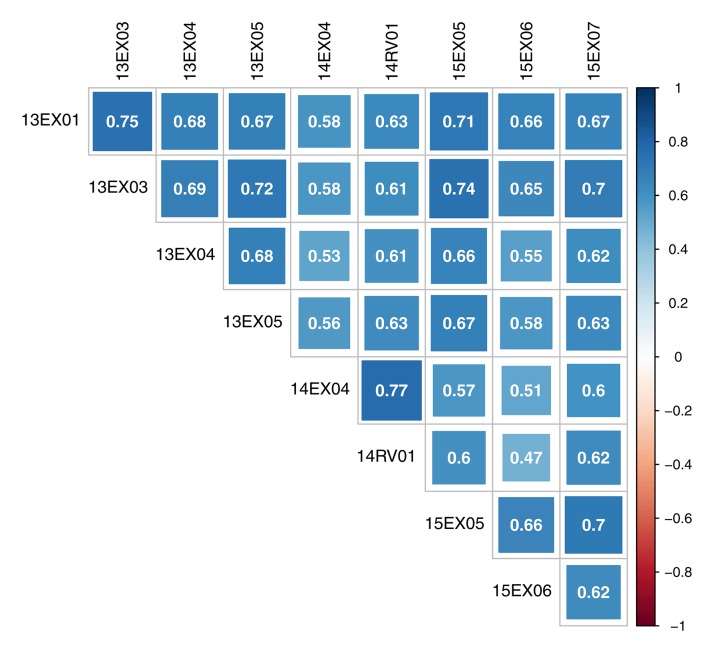
Correlation of oil content adjusted phenotype of hybrids between environments of the MET.

The oil content-adjusted phenotype of hybrids varied from 31.7 to 59.0% on the MET (see histograms in Supplementary Material, Figure S1). Hybrid-adjusted phenotypes were positively and significantly correlated between environments (Figure 2) with a minimum of 0.47, a maximum of 0.77 and average of 0.64. Intra-year correlations were slightly higher than between-year correlations, and environments observed in 2014 (14EX04, 14RV01) were less correlated with the two other year environments (13EX01, 13EX03, 13EX04 and 13EX05 sown in 2013, and 15EX05, 15EX06, and 15EX07 sown in 2015).

Using the three principal models of prediction (*GCA*, *FM* and *FMI* with all SNPs), we compared the REML variance components and their part of variance (Table 1). Female and male parts of variance were stable in the MET, despite visible differences in variance component values, particularly the environment 15EX07, implanted in Romany with a wider inter-row spacing (0.7 m) than the other environments (0.5 to 0.6 m). A decrease in the female part of variance and an increase in the male part of variance were observed in all environments using the correction based on the genomic relatedness of parents performed in the *FM* and *FMI* models. A significant female × male interaction (z-ratio equal to 2.71) was observed in a single environment (13EX05), and when included in the model, residual error was divided by 2. In all environments and for all models, the female part of genetic variance was superior to that of the male counterpart, showing roughly a ratio of (3/2) in favor of the female parent in the inheritance of hybrid genetic value for oil content.

**Table 1:** Number of observed hybrids (n.obs), mean oil content (in %), variance components and parts of variance (in %) [female, male, interaction female × male (inter.) and residual (resi.)] estimated using REML in *GCA*, *FM*, and *FMI* models, per environment (Env.).

**Model**	**Env.** **n.obs** **mean**	**13EX01** **272** **44.81**	**13EX03** **423** **45.26**	**13EX04** **407** **48.49**	**13EX05** **411** **41.43**	**14EX04** **418** **43.46**	**14RV01** **411** **40.72**	**15EX05** **459** **47.40**	**15EX06** **461** **45.45**	**15EX07** **458** **49.33**
**VARIANCE COMPONENTS**										
*GCA*	Female	2.74	1.68	1.44	2.00	4.19	3.70	2.60	1.58	6.12
	Male	1.44	0.67	0.75	0.61	1.66	1.74	0.88	0.73	2.91
	Resi.	2.09	1.03	1.03	1.40	1.79	1.70	1.40	1.40	4.00
*FM*	Female	2.13	1.21	1.07	1.55	3.22	2.90	1.84	1.22	4.88
	Male	1.50	0.77	0.78	0.69	1.79	1.83	1.09	0.83	3.23
	Resi.	2.08	1.03	1.03	1.39	1.79	1.70	1.40	1.40	4.00
*FMI*	Female	2.13	1.21	1.07	1.51	3.22	2.91	1.84	1.22	4.88
	Male	1.50	0.78	0.78	0.72	1.80	1.84	1.09	0.83	3.23
	Inter.	0.00	0.10	0.00	0.72	0.14	0.47	0.00	0.00	0.00
	Resi.	2.08	0.93	1.03	0.65	1.64	1.21	1.40	1.40	4.00
**PARTS OF VARIANCE**										
*GCA*	Female	0.44	0.50	0.45	0.50	0.55	0.52	0.53	0.43	0.47
	Male	0.23	0.20	0.23	0.15	0.22	0.24	0.18	0.20	0.22
*FM*	Female	0.37	0.40	0.37	0.43	0.47	0.45	0.42	0.35	0.40
	Male	0.26	0.26	0.27	0.19	0.26	0.28	0.25	0.24	0.27
*FMI*	Female	0.37	0.40	0.37	0.42	0.47	0.45	0.42	0.35	0.40
	Male	0.26	0.26	0.27	0.20	0.26	0.29	0.25	0.24	0.27
	Inter.	0.00	0.03	0.00	0.20	0.02	0.07	0.00	0.00	0.00

The three models described above were compared for their ability to predict unobserved hybrid genetic values on the same test sets (Table 2). Two sampling processes were experimented to estimate the reliability of GS either to complete the factorial design by predicting missing hybrids or to predict hybrids for which one or both parents were never observed by a descendant in the factorial design (the so-called T0 and T1 hybrids, (Technow et al., 2014)). The predictive ability of GS is high for oil content on the MET (0.783 in average for *FM* model) when the goal is to predict missing hybrids. The three models were nearly equally accurate, with only a 0.2% increase between the *GCA* model (the worse) and *FM* model (the best). The *FMI* model performed slightly better than the *FM* model in two environments (13EX05 and 14RV01), and these two environments had the greatest estimates of female × male interaction variances. The predictive ability is lower when the goal is to predict T0 or T1 hybrids with an average of 0.635 as the best performer (*FM* model). Once again, the *GCA* model was the least accurate model (0.575 in average), showing a 10% decrease in predictive ability compared to the *FM* model. The ranking between the *FM* and *FMI* models was similar to the previous sampling schema.

**Table 2:** Predictive ability of hybrid performances per environment (Env.) and average on the MET with *GCA*, *FM*, and *FMI* model BLUPs as the mean over the same 100 test sets (TS) using two sampling processes.

	**TS: any hybrids**			**TS: T1 or T0 hybrids**		
**Env**.	***GCA***	***FM***	***FMI***	***GCA***	***FM***	***FMI***
13EX01	0.756	0.762	0.761	0.580	0.653	0.651
13EX03	0.780	0.780	0.776	0.588	0.652	0.648
13EX04	0.767	0.768	0.766	0.572	0.641	0.639
13EX05	0.739	0.739	0.744	0.537	0.599	0.604
14EX04	0.835	0.836	0.835	0.589	0.665	0.665
14RV01	0.824	0.825	0.827	0.587	0.658	0.659
15EX05	0.800	0.800	0.799	0.596	0.634	0.633
15EX06	0.738	0.738	0.736	0.533	0.580	0.578
15EX07	0.796	0.797	0.796	0.590	0.635	0.633
Average	0.782	0.783	0.782	0.575	0.635	0.634

*T1 and T0 hybrids are hybrids for which one or both parents have no observed descendant in the training set*.

**Figure 3:**
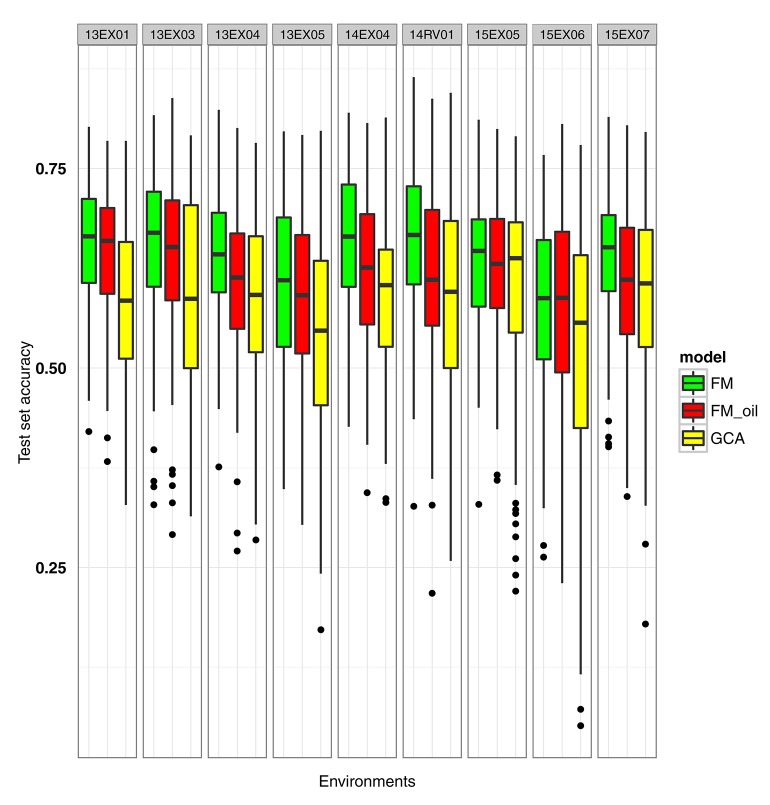
Boxplot of test set accuracy per environment for *GCA*, *FM* and *FM*_oil (*FM* modeling using knowledge of oil metabolic network) BLUPs. The model BLUPs were computed on the same 100 test sets. The test sets contained only T1 or T0 hybrids with untested parents.

Having observed that all methods are equally accurate to predict the missing hybrids of the factorial design, we focused on the prediction of T0 and T1 hybrids. Moreover, we made a prediction without considering the female × male interaction, as this interaction did not improve the accuracy and was CPU-time consuming. We attempted to improve the *FM* model by considering the genes involved in the oil metabolic pathway. Three hundred and seventy-two genes located throughout all chromosomes, having 3,746 non-redundant SNPs inside or 1,000 bp upstream and downstream, were considered (see details in Supplementary Material, Table S3). Our first attempt was to compute the female and male kinships involved in the *FM* model by considering only the 3,746 pre-selected SNPs. We named this model the *FM*_oil model. Boxplots of *GCA*, *FM* and *FM*_oil prediction accuracies for 100 random test sets of T0 and T1 hybrids are presented in Figure 3. *FM*_oil predictions were more accurate than *GCA* predictions, but the *FM* model was still the best, not only in mean, but it also showed a less variability in test set accuracies (Table 2).

By limiting the computation of parent relatedness to pre-selected oil SNPs, the *FM*_oil model is simplified and assumes that all important causal genes explaining oil content variability are already included in the considered metabolic pathway. To avoid an over-simplified assumption, we performed a multi-kernel model with two kinships for each parental effect, generated using pre-selected oil SNPs for one group and all remaining SNPs for the other group. This model assumes a different variance for each group of SNPs and each parental effect, leading to a more flexible model. With an average predictive ability in the MET of 0.628, this multi-kernel model slightly improved the average predictive ability of 0.612 for the *FM*_oil model but did not reach that of 0.635 for the *FM* model (Table 3). The *FM* model assumes that no interaction occurs between SNPs, neglecting the epistasis phenomena. We performed a multi-kernel BLUP model considering both the femalexfemale and the malexmale parts of the epistasis as a generalization of the additivexadditive epistasis modeling proposed by Su et al. (2012). With an average predictive ability of 0.623, this model did not improve the *FM* BLUPs (Table 3).

**Table 3:** Predictive ability of hybrid performances (mean over of 100 test sets and its variance) per environment (Env.) and in average on the MET with *GCA*, *FM*, *FM*_oil, mk_oil (multi-kernel *FM* model with two groups of SNPs) and mk_epi (multi-kernel model with female, male, female × female epistasis and male × male epistasis kernels) model BLUPs.

	**Mean**					**Variance**				
	***GCA***	***FM***	***FM*_oil**	**mk_oil**	**mk_epi**	***GCA***	***FM***	***FM*_oil**	**mk_oil**	**mk_epi**
13EX01	0.580	0.653	0.646	0.641	0.650	8.58 10^−3^	6.46 10^−3^	6.53 10^−3^	6.69 10^−3^	6.34 10^−3^
13EX03	0.588	0.653	0.642	0.645	0.645	1.55 10^−2^	1.01 10^−2^	1.17 10^−2^	1.02 10^−2^	9.91 10^−3^
13EX04	0.572	0.641	0.601	0.640	0.628	1.42 10^−2^	6.87 10^−3^	9.75 10^−3^	6.97 10^−3^	8.07 10^−3^
13EX05	0.537	0.599	0.579	0.594	0.575	1.71 10^−2^	1.19 10^−2^	1.25 10^−2^	1.17 10^−2^	1.43 10^−2^
14EX04	0.589	0.666	0.619	0.666	0.662	1.01 10^−2^	6.53 10^−3^	9.81 10^−3^	6.52 10^−3^	6.41 10^−3^
14RV01	0.587	0.659	0.616	0.656	0.632	1.61 10^−2^	1.04 10^−2^	1.34 10^−2^	1.08 10^−2^	1.09 10^−2^
15EX05	0.596	0.634	0.622	0.623	0.637	1.77 10^−2^	7.13 10^−3^	7.09 10^−3^	7.51 10^−3^	7.22 10^−3^
15EX06	0.533	0.580	0.574	0.564	0.555	2.61 10^−2^	1.20 10^−2^	1.48 10^−2^	1.38 10^−2^	1.88 10^−2^
15EX07	0.590	0.635	0.610	0.625	0.625	1.35 10^−2^	8.34 10^−3^	9.70 10^−3^	9.05 10^−3^	9.11 10^−3^
Average	0.575	0.635	0.612	0.628	0.623	1.54 10^−2^	8.86 10^−3^	1.06 10^−2^	9.25 10^−3^	1.01 10^−2^

*The model BLUPs were computed on the same 100 test sets. The test sets contained only T1 or T0 hybrids with parents never observed by their descendants*.

**Figure 4:**
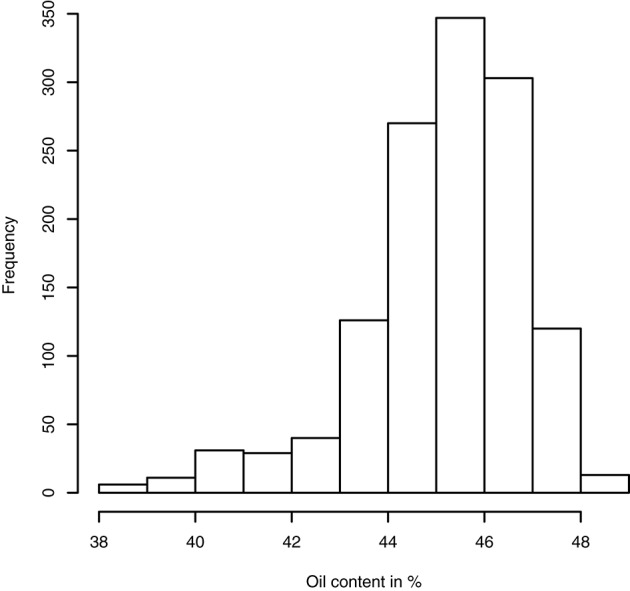
Histogram of hybrid mean predicted performance of oil content on the MET based on the mean of intra-environment ($\hat{\mu }$ + *FM* BLUP).

**Figure 5:**
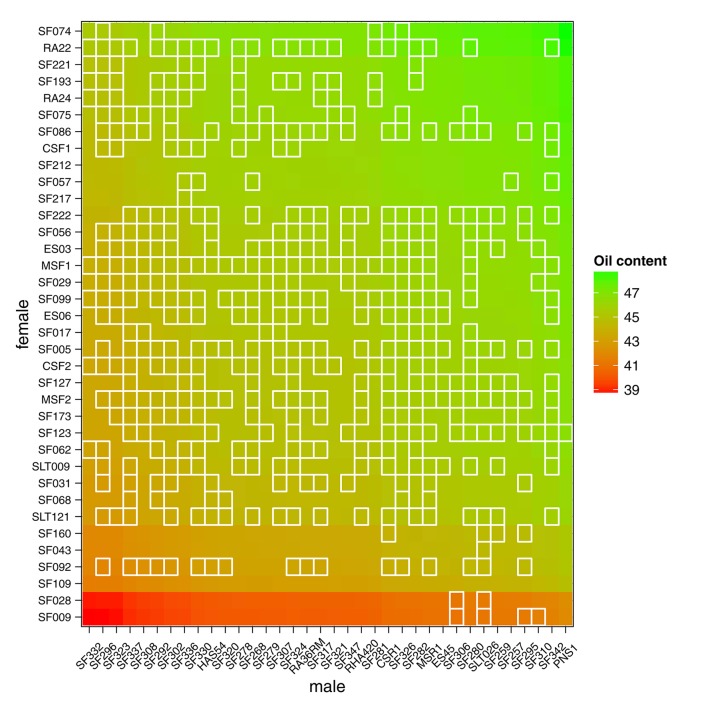
Mean predicted performance of hybrid oil content on the MET based on the mean of intra-environment ($\hat{\mu }$ + *FM* BLUP). Parents are ranked according to the mean of their descendants. Stable hybrids (Wricke's ecovalence less than 5) are surrounded with a blank square. A single hybrid is predicted as highly productive and stable in the right top corner of the heat map.

Having access to genetic value prediction of all hybrids in each environment of the MET with a high level of accuracy (0.783 in average for the *FM* BLUPs) facilitates selection of the best hybrids on average and affords an opportunity to examine their stability across environments. The distribution of hybrid mean predicted performance on the MET is shown in Figure 4. The least productive hybrid was predicted with a mean performance of 38.8%, and the most productive hybrid was predicted with a mean performance of 48.8% of seed oil content. Approximately 10% of hybrids had a predicted mean performance greater than 47%. To examine the stability of hybrids across environments, we computed the Wricke's ecovalence stability index (Wricke, 1962) using the hybrid predicted performances. This stability index measures how the hybrid predicted performances vary from an environment to an other. Figure 5 is a heat map representation of the mean predicted performance of hybrid with hybrids having a Wricke's ecovalence stability index (Wricke, 1962) less than 5, highlighted as a blank square. Hybrids predicted as producing a high oil content on average are generally not stable, only a single hybrid is predicted as stable in the right top corner of the heat map, its predicted mean performance and its Wricke's ecovalence were 48.3% and 4.83, respectively.

## 4. Discussion

As a starting point to evaluate the benefits of GS, in the present study, we compared the accuracy of hybrid performance predictions for seed oil content, a highly heritable breeding trait in sunflower. The simplest GCA-based model was compared with different genomic multi-kernel linear mixed models. We showed that the GCA-based model, ignoring parental pedigrees, is globally as accurate as more complex models to predict the oil content of unobserved sunflower hybrids in an incomplete factorial design where 36 maintainer lines (CMS form) were crossed with 36 restorer lines. This result reflects three main factors: (i) the accurate knowledge of the parental GCAs estimated in each environment from an average of at least 7 hybrid combinations, (ii) the strong additive effect of oil content in the MET, and (iii) the genetic distance between parents selected as unrelated to provide a good representation of the core collection studied in Cadic et al. (2013). However, there is an advantage to GS prediction (10% increase in accuracy) for hybrids of untested parents. Hybrids from untested parents are more distant from those observed than random missing combinations in the incomplete factorial design. Indeed, Hayes et al. (2009); Clark et al. (2012) indicated that it is more challenging to predict the values of unrelated genotypes and suggested that, in such situations, genomic predictions are more accurate than classical pedigree predictions.

GCA-based or GS predictions of missing hybrid performances is accurate in the MET (predictive ability of 0.78 on average), but with much less accuracy compared with Reif et al. (2013) (predictive ability of 0.97 by a leave-one-out hybrid cross validation). These two values are, in fact, not comparable as Reif et al. (2013) predicted the hybrid mean performances on the MET, whereas we predicted intra-environment hybrid performances. It is simpler to predict the mean performance compared with intra-environment performance, as the latter depends on the genetic by environment interaction, and therefore is more variable and less heritable. The lower a trait is heritable, the lower the GS predictive ability. However, intra-environment predictions are essential to access hybrid stability. Heffner et al. (2009) highlighted that GS is an important tool to address the challenge of genetic by environment interaction. Moreover, the lower the trait is heritable, the greater the prediction improvement expected from GS.

*FM* and *FMI* models differ by an interaction term that models parental allelic interaction or dominance. These models generally showed similar levels of accuracy in predicting untested hybrids or hybrids between untested parents. When their accuracies differed in one environment, a significant variance of the parental allelic interaction was observed, suggesting that only factors with sufficient variability could increase the accuracy of models including dominance compared to additive models. Moreover, a systematic small decrease of the *FMI* model accuracy compared to the *FM* model was observed when the variance component of this interaction was estimated as zero. The benefit of the inclusion of non-additive effects in an additive GS model is still subject to debate. Using simulations, Toro and Varona (2010) observed that inclusion of the dominance effect never decreased genetic gain in first generation selection in animal breeding programs whatever the ratio between additive and non-additive parts. Similarly, in a pig population, although Heidaritabar et al. (2016) showed no impact of dominance modeling in GS model accuracy for traits with a small ratio between additive and dominance, these authors did not observe any drawback. In contrast to these studies, the results of the present study are consistent with those of Reif et al. (2013) who observed small decreases in accuracy when dominance effects were included, depending on the traits and the intra or inter [B/R] group crosses. The significance of this decrease is important, but the lack of independence between the sampled test sets made it impossible to obtain a correct estimate of the variance of the mean accuracy necessary to build a test of significance. Neither the division by the square root of the number of sampled test sets nor the bootstrapped variance is correct with dependent results. Both methods provide a too small variance of the mean accuracy and thus conclude significance where there is no significance. Altogether, it might be assumed that the narrow-sense heritability of the trait plays an important role regarding the introduction of dominance effects in prediction models. As seed oil content is highly heritable (both narrow sense and broad sense), it is difficult to make a general conclusion. However, the high predictability of either *GCA* or *FM* models can explain why, without dense molecular scan and GS model, breeders have rapidly succeeded in transforming sunflower into a high valuable oil crop in the first half of the 20th century.

The use of biological information to enhance the accuracy of GS predictions was studied using simulations published by Pérez-Enciso et al. (2015). These authors showed that imprecision on QTL locations and non-exhaustive knowledge of all causal QTLs result in the rapid decline of the nearly perfect accuracy obtained when causal QTLs are all perfectly known. However, even with imperfect knowledge of 50% of genes, including causal QTLs, these authors showed a better accuracy compared to GS predictions with all SNPs. This encouraging result shows the interest of including functional knowledge in GS models. We tested the incorporation of biological knowledge on the oil metabolic network but we did not observe any improvement of the *FM* model predictions despite an improvement of the *GCA* model predictions. This finding is not surprising and is consistent with results of Spindel et al. (2016), who did not observe any improvement in accuracy with inclusion of historical GWAS results. Nevertheless, GS predictions using previous known genes involved in oil content metabolic network were better than the *GCA* model predictions (7% increase in average for hybrids of untested parents) with far less genotyping requirements than GBLUP predictions. Considering the phenotyping and genotyping efforts in breeding, this finding is an important practical result, showing that with SNPs on a limited number of genes in oil metabolism, we can accurately predict unknown hybrids without the need of either phenotyping both parents or genotyping them genome-wide. Accordingly, the prediction of traits of interest can be accessible for large panels by focusing on genes implicated in the trait using functional genomics knowledge and bioinformatics pipelines.

## 5. Conclusion

This study was conducted to compare the performance of classical prediction of hybrid based on the general combining aptitude (GCA) of their parents to current genomic predictions using whole genome sequencing. An incomplete factorial design of 36 maintainer lines (CMS form) crossed with 36 restorer lines, created during the course of the SUNRISE project, was used to estimate and compare accuracies of several hybrid predictions of seed oil content.

We showed that in such a design, classical GCA and GS predictions of hybrid performance had equal accuracy, as the GCA of each parent is well estimated for oil content, a highly heritable and mostly additive trait. However, predictions of hybrid performances of at least one untested parent are more accurate using GS models, showing that GS can accelerate the genetic gain by enabling better selection in hybrid panel of poorly known parent lines.

## Author contributions

BM and NL designed the study. BM performed the data analyses. FB, PP participated in the data analyses. NB, MB, GM, and EB provided genetic resources and phenotypic data. SC, LL, SM, and NP provided genomic data. LC provided metabolic data. PV designed the hybrid factorial design. BM, FB, LC, FV, PV, and NL drafted the manuscript. All authors have read and approved the manuscript.

### Conflict of interest statement

The authors declare that the research was conducted in the absence of any commercial or financial relationships that could be construed as a potential conflict of interest.
