# Supplementary material for: Genomic Prediction of Sunflower Hybrids Oil Content
Source: Front Plant Sci. 2017 Sep 21;8:1633. doi: 10.3389/fpls.2017.01633 (PMC5613134; doi:10.3389/fpls.2017.01633)
Supplement: Supplementary file 1 [file Presentation1.PDF]

*Supplementary Material*:
# Genomic prediction of sunflower hybrids oil content

## 1 SUPPLEMENTARY DATA

Data sheet S1: Oil genes of the metabolomic pathway

## 2 SUPPLEMENTARY TABLES AND FIGURES

### 2.1 Figures

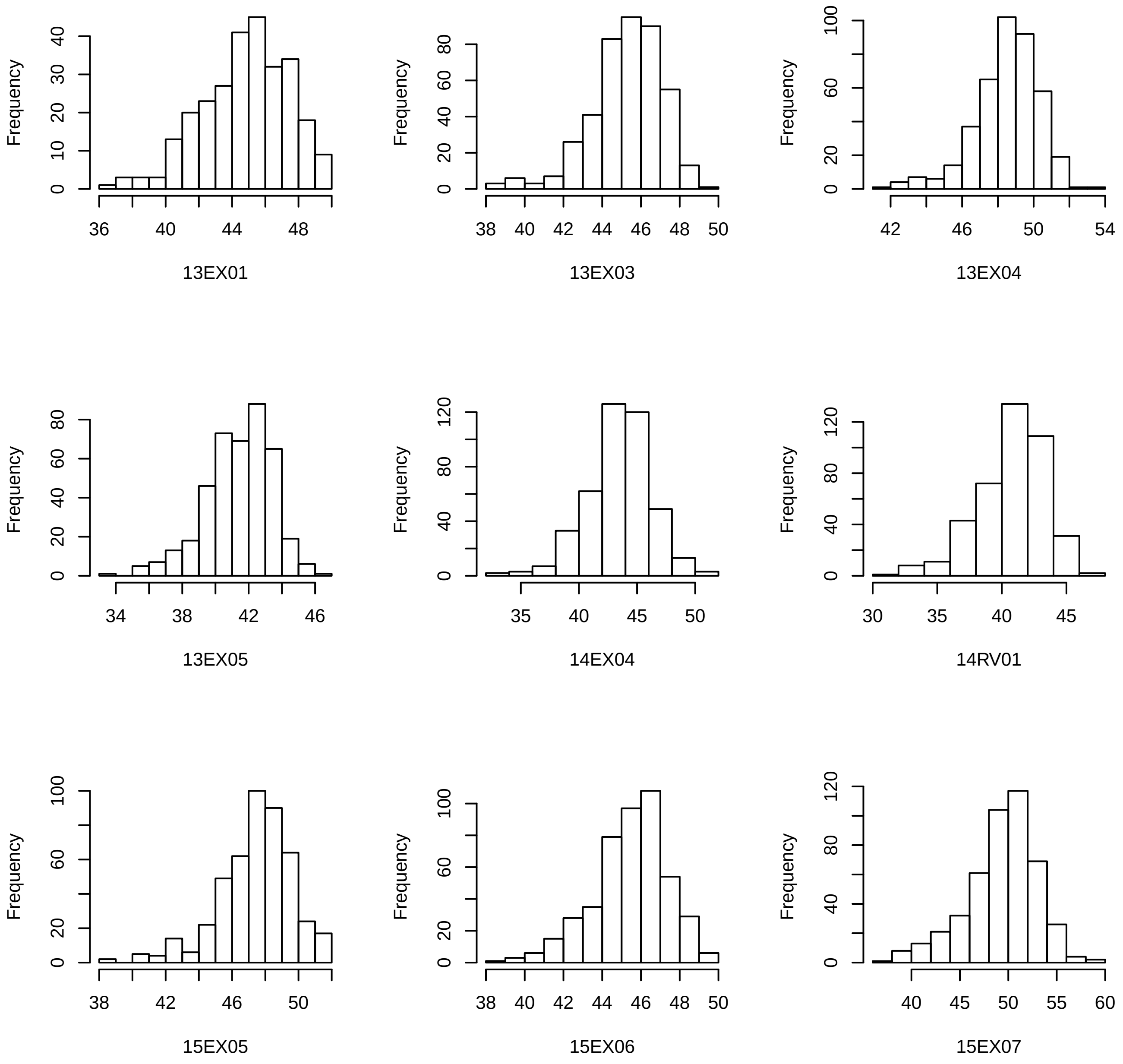


**Figure S1.** Histogram of oil content adjusted phenotype of hybrids per environment of the MET.

## 2.2 Tables

**Table S1.** Number of observed hybrids per female parent and environment.

| | 13EX01 | 13EX03 | 13EX04 | 13EX05 | 14EX04 | 14RV01 | 15EX05 | 15EX06 | 15EX07 |
|---|---|---|---|---|---|---|---|---|---|
| CSF1 | 8 | 11 | 12 | 11 | 13 | 13 | 13 | 14 | 14 |
| CSF2 | 8 | 11 | 12 | 11 | 12 | 12 | 13 | 13 | 11 |
| ES03 | 4 | 13 | 11 | 12 | 11 | 11 | 10 | 14 | 11 |
| ES06 | 7 | 12 | 11 | 12 | 13 | 13 | 14 | 13 | 14 |
| MSF1 | 5 | 13 | 13 | 11 | 13 | 12 | 13 | 13 | 12 |
| MSF2 | 7 | 13 | 13 | 12 | 13 | 13 | 14 | 14 | 14 |
| RA22 | 7 | 12 | 7 | 12 | 13 | 13 | 13 | 13 | 12 |
| RA24 | 5 | 9 | 10 | 9 | 10 | 9 | 13 | 12 | 10 |
| SF005 | 1 | 12 | 10 | 13 | 13 | 13 | 12 | 13 | 13 |
| SF009 | 9 | 13 | 11 | 12 | 12 | 11 | 15 | 15 | 15 |
| SF017 | 10 | 12 | 8 | 12 | 12 | 13 | 13 | 13 | 13 |
| SF028 | 12 | 13 | 13 | 13 | 14 | 14 | 14 | 14 | 14 |
| SF029 | 12 | 13 | 13 | 13 | 13 | 12 | 13 | 12 | 11 |
| SF031 | 5 | 11 | 11 | 10 | 8 | 8 | 13 | 14 | 14 |
| SF043 | 7 | 10 | 10 | 11 | 11 | 10 | 13 | 12 | 13 |
| SF056 | 6 | 11 | 9 | 11 | 11 | 11 | 12 | 12 | 12 |
| SF057 | 4 | 12 | 11 | 12 | 10 | 10 | 12 | 12 | 12 |
| SF062 | 10 | 13 | 13 | 12 | 13 | 13 | 13 | 11 | 13 |
| SF068 | 11 | 10 | 8 | 11 | 9 | 10 | 11 | 11 | 12 |
| SF074 | 8 | 11 | 11 | 10 | 10 | 10 | 13 | 12 | 12 |
| SF075 | 8 | 9 | 9 | 9 | 9 | 9 | 13 | 13 | 12 |
| SF086 | 10 | 13 | 12 | 13 | 12 | 13 | 14 | 14 | 13 |
| SF092 | 15 | 15 | 14 | 13 | 13 | 13 | 15 | 14 | 15 |
| SF099 | 3 | 8 | 5 | 6 | 5 | 2 | 7 | 6 | 7 |
| SF109 | 8 | 13 | 14 | 13 | 11 | 10 | 12 | 13 | 14 |
| SF123 | 3 | 11 | 12 | 11 | 13 | 13 | 11 | 14 | 13 |
| SF127 | 12 | 14 | 15 | 13 | 15 | 15 | 15 | 14 | 15 |
| SF160 | 3 | 12 | 12 | 11 | 12 | 12 | 13 | 11 | 12 |
| SF173 | 10 | 13 | 14 | 14 | 13 | 11 | 13 | 15 | 15 |
| SF193 | 10 | 12 | 12 | 11 | 10 | 11 | 14 | 14 | 15 |
| SF212 | 7 | 9 | 9 | 10 | 8 | 9 | 12 | 12 | 13 |
| SF217 | 7 | 10 | 11 | 11 | 11 | 11 | 13 | 13 | 11 |
| SF221 | 10 | 15 | 15 | 15 | 15 | 14 | 15 | 14 | 15 |
| SF222 | 7 | 12 | 12 | 11 | 12 | 12 | 11 | 12 | 12 |
| SLT009 | 8 | 11 | 12 | 10 | 13 | 13 | 12 | 13 | 13 |
| SLT121 | 5 | 11 | 12 | 10 | 12 | 12 | 12 | 12 | 11 |
| Mean | 7.56 | 11.75 | 11.31 | 11.42 | 11.61 | 11.42 | 12.75 | 12.81 | 12.72 |

**Table S2.** Number of observed hybrids per male parent and environment.

| | 13EX01 | 13EX03 | 13EX04 | 13EX05 | 14EX04 | 14RV01 | 15EX05 | 15EX06 | 15EX07 |
|---|---|---|---|---|---|---|---|---|---|
| CSR1 | 9 | 13 | 13 | 11 | 13 | 12 | 13 | 12 | 13 |
| ES45 | 6 | 13 | 12 | 13 | 13 | 13 | 14 | 12 | 12 |
| HAS54 | 2 | 5 | 5 | 5 | 13 | 13 | 12 | 12 | 12 |
| MSR1 | 1 | 3 | 4 | 5 | 8 | 10 | 12 | 14 | 14 |
| PNS1 | 5 | 10 | 9 | 9 | 10 | 10 | 13 | 12 | 12 |
| RA36RM | 3 | 13 | 13 | 11 | 11 | 11 | 13 | 13 | 13 |
| RHA420 | 11 | 10 | 15 | 10 | 15 | 15 | 14 | 14 | 14 |
| SF257 | 7 | 12 | 7 | 11 | 8 | 8 | 14 | 14 | 14 |
| SF259 | 8 | 14 | 13 | 13 | 11 | 11 | 14 | 10 | 13 |
| SF268 | 9 | 12 | 12 | 12 | 12 | 12 | 12 | 12 | 12 |
| SF278 | 9 | 12 | 11 | 13 | 12 | 11 | 14 | 13 | 13 |
| SF279 | 8 | 14 | 14 | 14 | 10 | 10 | 14 | 13 | 13 |
| SF280 | 7 | 13 | 13 | 11 | 12 | 12 | 13 | 13 | 13 |
| SF281 | 6 | 7 | 6 | 6 | 7 | 6 | 11 | 12 | 11 |
| SF282 | 6 | 10 | 11 | 11 | 10 | 11 | 10 | 11 | 12 |
| SF292 | 11 | 13 | 13 | 11 | 12 | 12 | 12 | 13 | 12 |
| SF295 | 8 | 12 | 12 | 11 | 12 | 12 | 12 | 12 | 12 |
| SF296 | 8 | 12 | 12 | 11 | 12 | 11 | 11 | 12 | 11 |
| SF302 | 9 | 12 | 12 | 12 | 10 | 10 | 12 | 12 | 12 |
| SF306 | 10 | 15 | 8 | 14 | 14 | 13 | 15 | 15 | 14 |
| SF307 | 12 | 14 | 10 | 13 | 14 | 12 | 13 | 14 | 13 |
| SF308 | 5 | 11 | 9 | 11 | 9 | 12 | 13 | 14 | 13 |
| SF310 | 9 | 13 | 13 | 13 | 13 | 13 | 13 | 13 | 13 |
| SF317 | 8 | 12 | 14 | 13 | 15 | 15 | 14 | 15 | 15 |
| SF320 | 7 | 12 | 13 | 13 | 13 | 13 | 13 | 13 | 13 |
| SF321 | 4 | 13 | 13 | 13 | 13 | 13 | 12 | 13 | 12 |
| SF323 | 10 | 12 | 12 | 12 | 12 | 12 | 11 | 12 | 10 |
| SF324 | 6 | 12 | 11 | 11 | 10 | 10 | 13 | 13 | 12 |
| SF326 | 10 | 15 | 14 | 14 | 14 | 14 | 14 | 14 | 15 |
| SF330 | 3 | 9 | 7 | 10 | 6 | 6 | 13 | 13 | 11 |
| SF332 | 13 | 14 | 15 | 14 | 15 | 14 | 14 | 13 | 15 |
| SF336 | 9 | 11 | 11 | 11 | 12 | 10 | 13 | 14 | 15 |
| SF337 | 9 | 12 | 13 | 13 | 12 | 12 | 13 | 13 | 12 |
| SF342 | 9 | 12 | 12 | 13 | 10 | 8 | 13 | 13 | 13 |
| SF347 | 6 | 14 | 13 | 13 | 14 | 13 | 11 | 13 | 13 |
| SLT026 | 9 | 12 | 12 | 10 | 11 | 11 | 11 | 10 | 11 |
| Mean | 7.56 | 11.75 | 11.31 | 11.42 | 11.61 | 11.42 | 12.75 | 12.81 | 12.72 |

**Table S3.** Caracteristics of gene involved in the oil metabolomic pathway: number of genes, total gene length in based pair (bp) and number of non-redondant SNPs (inside gene and within 1,000 bp upstream and downstream), per chromosome.

| | # genes | gene length (bp) | # SNPs |
|---|---|---|---|
| HanXRQChr01 | 27 | 102,330 | 423 |
| HanXRQChr02 | 23 | 114,860 | 327 |
| HanXRQChr03 | 13 | 64,839 | 133 |
| HanXRQChr04 | 15 | 86,990 | 171 |
| HanXRQChr05 | 32 | 78,685 | 190 |
| HanXRQChr06 | 11 | 29,167 | 57 |
| HanXRQChr07 | 11 | 77,733 | 71 |
| HanXRQChr08 | 21 | 80,424 | 258 |
| HanXRQChr09 | 18 | 84,722 | 175 |
| HanXRQChr10 | 47 | 230,881 | 375 |
| HanXRQChr11 | 18 | 119,608 | 247 |
| HanXRQChr12 | 22 | 86,462 | 228 |
| HanXRQChr13 | 30 | 108,276 | 167 |
| HanXRQChr14 | 25 | 90,142 | 172 |
| HanXRQChr15 | 20 | 100,222 | 186 |
| HanXRQChr16 | 22 | 120,178 | 289 |
| HanXRQChr17 | 17 | 81,006 | 277 |
| Total | 372 | 1,656,525 | 3,746 |
